# The Significance of the Treatment for Elderly Severe Trauma Patients Who Required Intensive Care

**DOI:** 10.7759/cureus.39110

**Published:** 2023-05-16

**Authors:** Yusuke Sawada, Yuta Isshiki, Yumi Ichikawa, Kazunori Fukushima, Yuto Aramaki, Kei Kawano, Mizuki Mori, Kiyohiro Oshima

**Affiliations:** 1 Department of Emergency Medicine, Gunma University Graduate School of Medicine, Maebashi, JPN

**Keywords:** activities of daily life, physical status, cost, trauma, elderly

## Abstract

Purpose

Elderly trauma patients have a higher risk of severe disability and death, and this outcome burden in elderly trauma patients must be addressed in countries in which the population is aging. The clarification of the unique clinical features of elderly people who have experienced trauma is important. The purpose of this study is to evaluate the significance of the treatment for elderly severe trauma patients based on the prognosis and total hospital cost.

Methods

Trauma patients transferred to our emergency department (ED) and admitted to our intensive care unit (ICU) directly or through emergency surgery between January 2013 and December 2019 were examined. We divided patients into three groups: <65 years old (Group Y); 65-79 years old (Group M); and ≥80 years old (Group E). We compared the pre- and post-trauma American Society of Anesthesiology Physical Status (ASA-PS) score and the Katz Activities of Daily Living (ADL) questionnaire at arrival among the three groups. In addition, the duration of ICU and hospital stays, hospital mortality, and total treatment costs were compared.

Results

There were 1,652 patients admitted to ICU through the ED from January 2013 to December 2019. Of those patients, 197 trauma patients were analyzed. There was no significant difference in injury severity scores between the groups. Significant differences in both the ASA-PS and Katz-ADL scores in posttrauma status were observed among the three groups (posttrauma ASA-PS, 2.0 (2.0, 2.8) in Group Y, 3.0 (2.0, 3.0) in Group M, 3.0 (3.0, 3.0) in Group E, p < 0.001*, posttrauma Katz-ADL, 10.0 (3.3, 12.0) in Group Y, 5.5 (2.0, 10.0) in Group M, 2.0 (0.5, 4.0) in Group E, p < 0.001). The duration of both ICU and hospital stay was significantly longer in Group E compared to the other groups (ICU stay, 4.0 (3.0, 6.5) days in Group Y, 4.0 (3.0, 9.8) days in Group M, 6.5 (3.0, 15.3) days, p = 0.006, hospital stay, 16.9 (8.6, 33.0) days in Group Y, 26.7 (12.0, 51.8) days in Group M, 32.5 (12.8, 51.5) days in Group E, p = 0.005). ICU and hospital mortality were highest in Group E compared with the other groups, but the differences were not significant. Finally, the total hospital cost in Group E was significantly higher than the other groups.

Conclusions

In elderly trauma patients who required intensive care, PS and ADL in posttrauma status were worse, ICU and hospital stays were longer, and ICU and hospital mortality were higher compared with younger patients. In addition, medical costs were greater in elderly patients. It is supposed that the therapeutic effect observed in young trauma patients cannot be expected in elderly trauma patients.

## Introduction

Traditionally, trauma has been viewed as a leading cause of disability and death in patients aged ≤45 years [1,2]. However, approximately 500,000 elderly patients are admitted to trauma centers after injury annually in the United States [3]. In Japan, deaths caused by trauma are typically classified as ‘unexpected accident’. ‘Unexpected accident’ is the seventh leading cause of death over the lifespan in Japan [4]. Therefore, in Japan, it is difficult to classify trauma as a leading cause of death. However, in 2020, more than 30,000 Japanese aged ≥65 years died by ‘unexpected accident’ [4]. In addition, trauma injuries such as fractures and tumbles (that means to fall down by a tangle of legs or a stumble) are the fourth leading reason for requiring nursing care in Japanese people aged ≥65 years old [5]. Thus, elderly trauma patients have a higher risk of severe disability and death compared to similar trauma patients <65 years old [6,7]. This outcome burden in elderly trauma patients must be addressed in countries in which the population is aging, and it is important to clarify the unique clinical features of elderly people who have experienced trauma compared to those of younger trauma patients.

We hypothesized that the prognosis and post-trauma general states are more severe, and the total hospital cost is higher in elderly severe trauma patients. The aim of this study was to evaluate clinical features, including the pre- and post-trauma activity of daily life (ADL) and medical costs, in elderly trauma patients who required intensive care and compare the findings with those of younger trauma patients.

## Materials and methods

The research ethics board of the Gunma University Hospital approved this retrospective study (HS2020-005) without the necessity for informed consent. The execution of this study was publicized on the homepage of Gunma University.

Trauma patients transferred to our emergency department (ED) and admitted to the ICU of Gunma University Hospital directly or through emergency surgery between January 2013 and December 2019 were studied. We included trauma patients aged 18 and over. Exclusion criteria were patients with cardiac arrest on arrival, patients without positive requests from the family about active treatment, patients in the terminal stage of malignant diseases or liver cirrhosis, and patients moved from ICU to the normal ward within 24 hours. We divided patients into three groups based on their age: <65 years old (Group Y); 65-79 years old (Group M); and ≥80 years old (Group E). In general, elderly people are regarded as those aged ≥65 years [8,9], and the average life expectancy at birth in developed countries is ≥80 years [10]. We compared pre- and post-trauma general states using the American Society of Anesthesiology-Physical Status (ASA-PS) scoring [11] and the Katz ADL questionnaire [12] among the three groups. Pre-trauma general states were evaluated based on information from the patients and/or the patients’ relatives after admission to our hospital, and those of post-trauma were evaluated at discharge from our hospital. In addition, the abbreviated injury scale (AIS), the injury severity score, the revised trauma score (RTS), and the trauma and injury severity score (TRISS; as a predictor of the probability of death) at arrival were compared among the three groups. The duration of ICU and hospital stays, hospital mortality, and total medical costs were also compared. Those data were obtained from electronic medical charts.

Statistical analysis

The distribution of normality was evaluated with the Shapiro-Wilk test. Those that fit the normal distribution were expressed as mean ± standard deviation, while those that did not fit were expressed as median and interquartile range. Categorical variables were expressed as values and/or percentages. The Kruskal-Wallis test was used for the comparisons of continuous variables among the three groups. Post-hoc analyses were subsequently conducted to evaluate the differences between the three groups. Comparisons of categorical variables were done with the chi-squared test. A p-value of less than 0.05 was considered statistically significant. The Statistical Product and Service Solutions (SPSS) Statistics ver. 26.0 (IBM Corp., Armonk, NY, USA) software was used for the statistical analysis.

## Results

**Figure 1:**
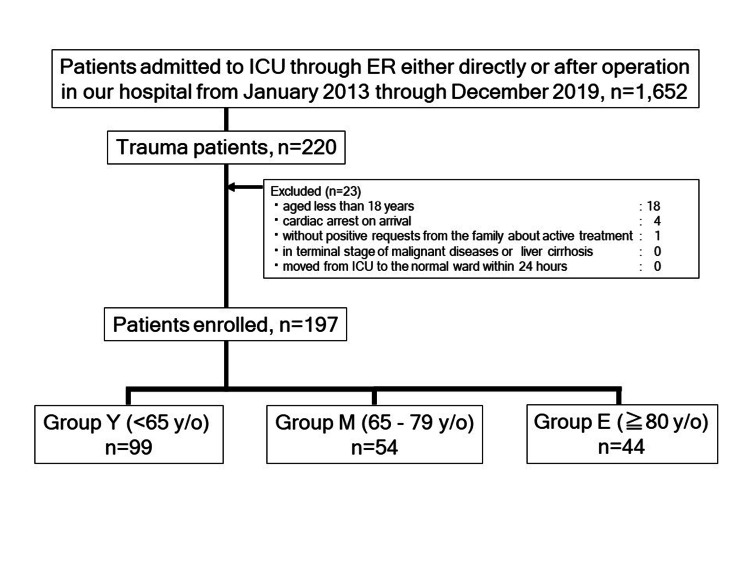
Study flowchart ICU: intensive care unit, ER: emergency room, y/o: years old

From January 2013 to December 2019, 1,652 patients were admitted to ICU through the ED. Of those patients, 197 patients with trauma were analyzed in the study (Figure 1).

The causes of trauma were as follows; traffic accident in 99 patients, fall in 54 patients, penetration in 13 (all were stub wounds and there was no gunshot wound), tumble in 12, caught by machinery in eight, ski or snowboard injury in six, and other causes in five patients. Blunt and penetrating trauma were 184 and 13, respectively. The median age in all 197 patients (145 male, 52 female) was 64 years (ranging from 18 to 92) years old. Pre- and post-trauma (evaluation at discharge from our hospital) activities, severities of trauma, duration of ICU and hospital stay, hospital mortality, and total costs in all 197 patients are shown in Table 1 ($1 = 138.59 Japanese yen).

**Table 1:** Characteristics of all patients evaluated in this study ASAPS: American Society of Anesthesiology-Physical Status; ADL: activities of daily living; AIS: abbreviated injury scale; ISS: injury severity score; RTS: revised trauma score; TRISS: trauma and injury severity score Data are presented as the median and interquartile ranges (IQR) for age, ASA PS, Katz ADL score, AIS, ISS, RTS, TRISS, duration of ICU and hospital stays, and total costs in our hospital. Other parameters are expressed as values and/or percentages.

Variable	
Age (years)	64 (42-78)
Male/Female	145/52
ASA PS score	
pre-trauma	2 (1-2)
post-trauma	2 (2-3)
Katz ADL score	
pre-trauma	12 (12-12)
post-trauma	5 (1-10)
AIS	
Head/neck	0 (0-4)
Face	0 (0-1)
Chest	1 (0-3)
Abdomen	0 (0-3)
Extremities	0 (0-2)
Skin/general	0 (0-0)
ISS	18 (14-25)
RTS	7.5500 (6.3756-7.8408)
TRISS	0.0668 (0.0275-0.2423)
The duration of ICU stay (days)	4 (3-9)
The duration of hospital stay (days)	21 (11-43)
The ICU mortality	2.5% (5/197)
The hospital mortality	7.1% (14/197)
The total costs in our hospital ($)	12235 (7049-23158)

Table 2 shows the characteristics of the three groups. Regarding the trauma causes, traffic accident was around 50% and was the greatest cause in all three groups. On the other hand, the rate of tumble was high in Groups M and E compared with that in Group Y, and trauma caused by being caught in machinery, skiing/snowboarding, and equitation was not observed in Group E. Thus, there was a significant difference in the causes of trauma among the three groups. There were no significant differences in each AIS of the six anatomical regions and ISS. RTS in Group E was the most severe among the three groups without a significant difference, and TRISS (the predicted mortality rate) was significantly higher in Group E than in Groups Y and M. As shown in Table 2, there were significant differences in pre- and post-trauma ASA-PS and Katz-ADL scores among the three groups.

**Table 2:** Comparison of characteristics among the three groups AIS: abbreviated injury scale; ISS: injury severity score; RTS: revised trauma score; TRISS: trauma and injury severity score; ASAPS: American Society of Anesthesiology-Physical Status; ADL: activities of daily living. Data are presented as the median and interquartile ranges (IQR) for age, ASA PS, Katz ADL score, AIS, ISS, RTS, TRISS, duration of ICU and hospital stays, and total costs in our hospital. Other parameters are expressed as values and/or percentages. In the causes of trauma, the number in the parenthesis is the sum of each cause of trauma. *: p<0.05

	Group Y (n=99)	Group M (n=54)	Group E (n=44)	p value
Age	42.0 (28.5-54.5)	71.0 (68.0-75.8)	83.0 (82.0-86.0)	<0.001*
Male (real number (%))	81 (82%)	34 (63%)	30 (68%)	0.026*
The causes of trauma				0.015*
Traffic accident (99)	44 (44.4%)	33 (61.1%)	22 (50.0%)	
Fall (54)	28 (28.3%)	10 (18.5%)	16 (36.4%)	
Penetrating (13)	10 (10.1%)	3 (5.6%)	0	
Tumble (12)	2 (2.0%)	4 (7.4%)	6 (13.6%)	
Caught by machinery (8)	5 (5.1%)	3 (5.6%)	0	
Ski・Snowboard (6)	6 (6.1%)	0	0	
Others (5)	4 (4.0%)	1 (1.8%)	0	
AIS				
Head/neck	0 (0-4.0)	2.5 (0-4.0)	0.5 (0-4.0)	0.421
Face	0 (0-1.0)	0 (0-1.0)	0 (0-0)	0.066
Chest	1 (0-3.0)	0 (0-3.0)	3.0 (0-3.0)	0.120
Abdomen	0 (0-3.0)	0 (0-2.8)	2.0 (0-3.0)	0.322
Extremities	0 (0-2.0)	0 (0-2.8)	0 (0-2.0)	0.744
Skin/general	0 (0-0)	0 (0-0)	0 (0-0)	0.535
ISS	19 (14-25)	17 (14-26)	21 (14-28)	0.686
RTS	7.8408 (6.8174-7.8408)	7.5500 (6.3756-7.8408)	6.9040 (5.9672-7.8408)	0.133
TRISS	0.03047 (0.01141-0.08837)	0.12213 (0.05677-0.23286)	0.23613 (0.07609-0.43379)	<0.001*
Pre-trauma				
ASA-PS	1.0 (1.0-1.0)	2.0 (1.0-3.0)	2.0 (2.0-2.3)	<0.001*
Katz-ADL	12.0 (12.0-12.0)	12.0 (12.0-12.0)	12.0 (12.0-12.0)	<0.001*
Post-trauma (at discharge)				
ASA-PS	2.0 (2.0-2.8)	3.0 (2.0-3.0)	3.0 (3.0-3.0)	<0.001*
Katz-ADL	10.0 (3.3-12.0)	5.5 (2.0-10.0)	2.0 (0.5-4.0)	<0.001*

**Figure 2:**
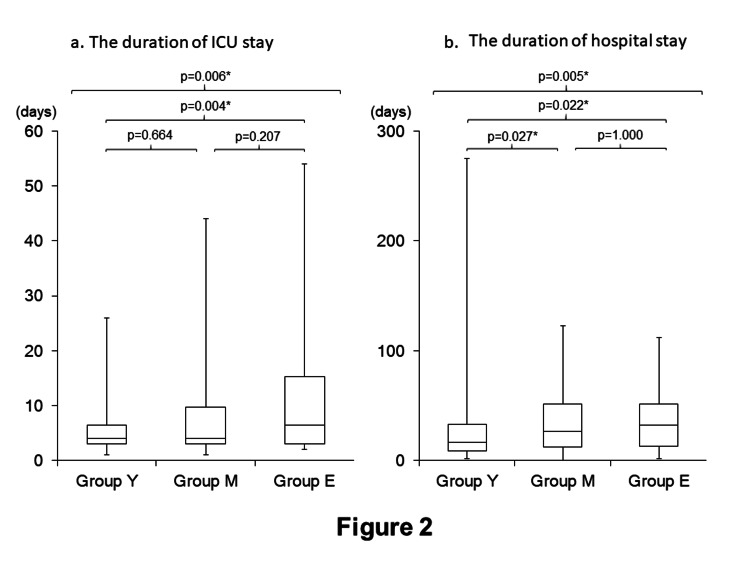
The comparisons of the duration of ICU stay and hospital stay a. The comparison of the duration of ICU stay. ICU: intensive care unit b. The comparison of the duration of hospital stay. *: p<0.05

Figure 2 examines the duration of ICU (Figure 2a) and hospital stays (Figure 2b; the duration of stay in our hospital). There were significant differences in the duration of both ICU and hospital stays between the groups, and those in Group E were the longest among the three groups (the duration of ICU stay: Group Y, 4.0 (3.0-6.5) days; Group M, 4.0 (3.0-9.6) days; and Group E, 6.5 (3.0-15.3) days) (the duration of hospital stay: Group Y, 16.9 (8.6-33.0) days; Group M, 26.7 (12.0-51.8) days; and Group E, 32.05 (12.8-51.5) days).

**Figure 3:**
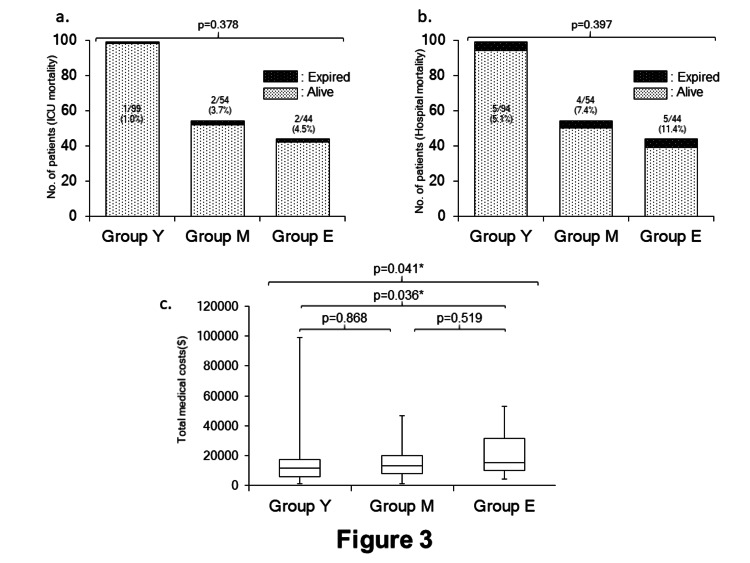
The comparisons of the ICU mortality, the hospital mortality, and the total hospital costs a. The comparison of the ICU mortality among the three groups. Numbers on or in each bar are shown as the number of ICU death/total patients’ number in each group (the ICU mortality). ICU: intensive care unit b. The comparison of hospital mortality among the three groups. Numbers on or in each bar are shown as the number of hospital death/total patients’ number in each group (the hospital mortality). c. The comparison of the total hospital costs. *: p<0.05

There were no significant differences in the ICU and hospital mortalities among the three groups, however, those in Group E were the highest of the three groups (Figure 3a, Figure 3b). The total medical cost (during the stay in our hospital) of Group E was significantly higher than Groups Y and M (Group Y, $11508 (5941-17621); Group M, $13439 (7955-20143); and Group E, $15478 (10217-31503)) (Figure 3c).

## Discussion

It has been already reported that the prognosis including mortality and disability in elderly trauma patients becomes worse compared with their younger counterparts [13-16], and our results in this study mostly coincide with those previous studies.

Traffic accident was the most common cause of injury in all three patient groups in this study. Fall was the second most common cause in all groups, however, the rate of fall in Group E was higher than in Groups Y and M. In addition, the rate of tumble in the elderly group was higher than that of the other two groups. Elderly patients exhibit different injury patterns compared to their younger counterparts [17], and it has been already reported that fall is the most common mechanism of injury and the leading cause of trauma-related deaths in geriatric trauma patients [18,19]. Most falls are ground-level in the frail elderly, however, 14% of falls were from high levels (stairs, ladders, and roofs) in the active elderly [20]. All level falls can cause devastating and life-altering injuries [18,21], therefore, the prevention of fall and tumble is important, especially for elderly people.

In this study, the median of ISS in all three groups was more than 15 and similar. Severe trauma is generally defined as ISS>15 [22,23], therefore, the subjects in the present study were severe trauma patients. The RTS in the elderly group was the most severe among the three patient groups, but the differences were not significant. TRISS in the elderly group was significantly higher than in the other groups. The value of TRISS is dependent on whether the age of the individual is 55 years or less. Group Y in this study included 75 patients (75.8%) aged ≤55 years old. Therefore, age might affect our results in some measure. However, our results do suggest that the impact of trauma on general conditions such as blood pressure, consciousness, and respiratory rate, which are included as parameters to calculate RTS and TRISS, is more serious in elderly patients compared with younger patients with similar degree trauma. The physiologic reserve is generally deteriorated with aging [19,24], and our results coincide with those findings.

The physical status and ADL of the elderly patients in the pre-trauma status were poor, and, at discharge from our hospital, physical status and ADL have remarkably deteriorated in elderly trauma patients. The durations of ICU and hospital stay were longer in the elderly trauma patients and mortalities in ICU and hospital in the elderly trauma patients were worse than that of the younger trauma patients. In the elderly population, the decreased functional residual capacity and increase comorbidity may increase the risk of poor clinical outcomes. Antihypertensive medications, antiarrhythmic medications, and anticoagulant medications also impair the physiological presentation when injury occurs. In fact, the Eastern Association for the Surgery of Trauma published their guidelines for the management of elderly trauma patients in 2012, describing that pre-existing conditions and/or severe anatomic injuries dramatically increase the risk of poor outcomes in elderly patients, and concluding that evidence-based care of this population requires aggressive triage, correction of coagulopathy and limitation of care when clinical evidence suggests a clear likelihood of poor prognosis [25]. Bonne et al. also insisted that early recognition of injury, even minor, and expedited care using specialized teams will help to improve outcomes for older adult trauma patients [26]. However, to put it the other way around, the treatment of elderly severe trauma patients implies treating aggressive patients with a limited physiological reserve and uncertain outcomes, and elderly patients with severe trauma have a limited response to injury [6]. Taking those reports and our results into consideration, it is supposed that the therapeutic effect observed in young trauma patients cannot be expected in elderly trauma patients. Recently, there are data demonstrating the improved value of palliative care and hospice at the end of life [27], and studies that specifically involve geriatric trauma patients are also reported [28-30]. A timely palliative care consultation for a vulnerable patient population may be required [28].

The results of this study also showed that it costs more to treat elderly trauma patients compared to younger trauma patients. Rehabilitation after trauma might improve PS and ADL somewhat, however, it takes longer time and more resources to continue rehabilitation. It is difficult to predict who will or should receive quantitatively futile care, however, Fleischman et al. estimated that the costs associated with futile care in geriatric trauma patients have a median cost of $87,391 compared with non-futile care that had a median cost of $33,373 [31]. In Japan, patients aged ≥75 years old with low-income pay only 10% of the total medical charges, and the remainder is paid by the country. In fact, the burden of medical charges to the country increases every year, and national medical care expenses in Japan are currently $304.5 billion USD. Increasing medical charges are a large burden on the national finances of Japan, along with the added costs of the coronavirus disease 2019 pandemic, and have become a significant social problem. The prevention of trauma in elderly people is important because the causes of trauma in elderly people are limited to some extent. Kozar et al. also reported the importance of effective prevention strategies as the proposed solution to falls in geriatric trauma [19]. This issue should be addressed in societies with an elderly population that is increasing.

Limitations

This study was retrospectively performed at only one institution, and the number of patients analyzed in this study was not very large. Evaluation of long-term prognosis, including the post-rehabilitation status, such as PS and ADL, and all costs after discharge from our hospital should be performed. Multicenter studies are warranted to clarify further the appropriate treatment for elderly severe trauma patients.

## Conclusions

In elderly trauma patients requiring intensive care, PS and ADL in post-trauma status are remarkably worse, ICU and hospital stays are longer, ICU and hospital mortality are higher, and more medical costs are necessary compared with that in younger trauma patients. It is supposed that the therapeutic effect observed in young trauma patients cannot be expected in elderly trauma patients. The establishment of effective strategies to prevent elderly trauma is urgent in aging societies such as Japan.
